# 

**Published:** 1913-08-23

**Authors:** 


### Appointments.

The following appointments have been made at the Leicester Royal Infirmary:—Mr. L. T. Challenor, M.R.C.S., L.R.C.P., second house surgeon, appointed senior house surgeon; Mr. S. H. Wilkinson, M.B., Ch.B., appointed second house surgeon; Mr. N. P. Laing, M.B., Ch.B. (Liverpool), appointed house physician; Mr. W. P. Hogg, M.B., Ch.B. (Aberdeen), appointed assistant house surgeon; Mr. C. J. Timms, L.R.C.P. & S. (Edinburgh), appointed assistant house physician.

### Obituary.

Dr. Robert Arthur Simpson, of Thornton Heath, died suddenly in Grange Wood Park recently, while returning home from visiting his patients. He had graduated at Glasgow in 1874, was at one time a Navy surgeon, and had been living at Thornton Heath for nearly twenty years.

Surgeon-Colonel J. Richardson, Honorary Physician to the King, has died at Totland, aged seventy-six. Formerly in the Indian Medical Service, Surgeon-Colonel Richardson served in the Bhootan Expedition, 1864-5-6, being present at the capture of the Bala Pass. He retired in 1889.

### Buildings Contemplated.

Ballymena.—The Guardians have decided to take up a loan of £7,500 for a new workhouse hospital.

Barnet.—A recommendation by the infirmary committee that a new infirmary to contain 192 beds should be built at a cost of not more than £80 per bed has been adopted by the Guardians.

Billericay.—The Rural District Council have decided to apply to the Local Government Board for sanction to borrow £5,643 for a new hospital.

Bradford.—The Health Committee has recommended the Corporation to build a smallpox hospital with accommodation for twelve beds.

Brigg.—The Local Government Board has approved amended plans for a new infirmary estimated to cost £7,490.

Budapest.—It is announced in a Vienna journal that the municipal authorities of Budapest have approved the proposals submitted by the Mayor for the extension of the St. Gellert and St. Laszlo Hospitals. The cost of the extensions is estimated at £189,400.

Caistor.—The Rural District Council is about to apply to the Local Government Board for sanction to borrow £1,800 for the enlargement of the isolation hospital.

Chichester.—The West Sussex County Council has decided to spend £4,700 upon additions to the County Mental Hospital, including an engine-house and engine.

Croydon.—The Corporation has decided to apply to the Local Government Board for sanction to borrow a further £1,020 for additional accommodation for phthisis cases.

Eastbourne.—Plans and an estimate of £5,075 for a new workhouse have been approved by the Guardians.

Hebden.—The Bradford Corporation Health Committee has instructed the city architect to prepare plans for a sanatorium at Hebden, near Glassington, to accommodate 150 beds.

Huddersfield.—The Insurance Committee have approved plans for a sanatorium at a cost of about £13,000.

Killingbeck.—The Leeds Corporation has decided to provide a temporary consumptive hospital at Killingbeck.

Marlow.—The Charity Commissioners have sanctioned the building of a new hospital. The site has been given, and, of the £2,000 required, £1,500 has already been promised.

Middlesbrough.—The Middlesbrough Sanitary Committee has accepted an estimate of £150 for a dispensary in Grange Road.

Rotherham.—The Corporation has under consideration the question of providing a smallpox hospital at the Ulley reservoir.

Shrewsbury.—The Salop County Council has decided to provide thirty additional beds at the Shirlett Sanatorium at an estimated cost of £2,227. Application will be made to the Local Government Board for a grant of £1,336 towards the cost.

Sydney (New South Wales).—It is proposed to alter and extend the Sydney Hospital at an estimated cost of £237,901.

West Riding.—Plans have been approved for the erection, by the West Riding County Council, of a sanatorium on a site comprising about 140 acres in the valley of the Wharfe, opposite Ben Rhydding. Accommodation for 100 beds will be provided to commence with.

Witton.—The Birmingham Corporation has decided to enlarge the hospital at Witton.

### Gifts and Bequests.

Mrs. Harriet Eleanor Freer, of Sidmouth, has left £100 to the Sidmouth Cottage Hospital.

Miss Emily Howard, of Canterbury, has left £300 to the Westminster Hospital, £200 to the Kent and Canterbury Hospital, and £100 to the Canterbury Dispensary.

Mr. Joseph Cobb, of Hounslow, has left £500 to the Butchers' Charitable Institution, and, subject to his wife's interest, £500 to the Hounslow Hospital and £250 to the Hospital for Incurables.

The Leicester Royal Infirmary has received intimation of the following bequests :—£500 free of duty under the will of the late Mr. Walter Beales Clark, a generous benefactor of the institution in his lifetime; £100 free of duty under the will of the late Miss Helen Tomblin; a third share in the residuary estate of the late Mr. Samuel Bonnett Hill, of Ashby-de-la-Zouch; a half-share of the proceeds of sale of her house under the will of the late Miss Elizabeth C. Ward, of South Croxton.

Miss Maria Mercer, of Clayton-le-Moors, Lancashire, who has left charitable gifts amounting to £153,000, has bequeathed £5,000 to the Blackburn and East Lancashire Infirmary; £3,000 to the Victoria Hospital at Accrington; £2,000 to Dr. Barnardo's Homes; £1,000 each to the Children's Home and Orphanage, Edgworth, the Blackburn and District Orphanage, Wilpshire, Muller's Orphanage, Bristol, the Railway Benevolent Institution, and the Railway Servants' Orphanage, Derby. The testatrix left the residue of her estate for such charitable purposes in England as the trustees of her will shall determine. The amount available for charitable purposes under Miss Mercer's will is stated to be more than £130,000.

### THE HIGHLANDS AND ISLANDS MEDICAL SERVICE.

The names of those who are to constitute the Board to administer the Highlands and Islands Medical Service Act were announced in Parliament last week as follows: Sir John Dewar, chairman; Sir Donald MacAlister, Principal of the University of Glasgow; Lady Susan Gilmour, honorary secretary of the Queen Victoria Jubilee Institute for Nurses; Dr. McVail, of the Scottish Insurance Commission; Dr. Leslie Mackenzie, of the Local Government Board; Dr. John Macpherson, of the Lunacy Board; Mr. J. L. Robertson, Chief Inspector under the Scotch Education Department.

### SEA-GOING HOLIDAYS.

The Orient Line of mail steamers to Australia provide an attractive holiday sea-trip of fifteen days, visiting Gibraltar and Toulon. The homeward mail steamer connects with the outward, and passengers can either make a tour of six days in Southern Spain and Morocco or by proceeding to Toulon have a short stay on the Riviera. The trip is made by the 12,000-ton vessels of the Line, the accommodation on board of which is described as "palatial" in character.—An interesting and well illustrated booklet by Mr. Charles Hussey dealing with this holiday sea-trip has just been issued by the Orient Company, and can be obtained on application at the Company's Offices, 5 Fenchurch Avenue, London, E.C., and 28 Cockspur Street, London, S.W.

Cheap Summer Excursions.—The last of the cheap summer excursions inaugurated this year by the Paris, Lyons, and Mediterranean Railway will leave London for Auvergne, Dauphiny, Savoy, Provence, and Corsica on August 30.

